# Characterization of the Adverse Drug Reactions Associated with Psychotropic Medications Based on a Spontaneous Reporting Systems Database: A Retrospective Analysis

**DOI:** 10.2147/RMHP.S578820

**Published:** 2026-04-01

**Authors:** Yassin Eltorki, Oraib Abdallah, Zeana Alkudsi, Waad Elamin, Ahmed Gharaibeh, Mariam Mustafa, Noor Abu Shameh, Sadaf Riaz, Islam Mahran, Noriya Al-Khuzaei, Ovais Wadoo, Monica Zolezzi

**Affiliations:** 1 Pharmacy Department, Mental Health Services, Hamad Medical Corporation, Doha, Qatar; 2 Pharmacy Department, Al Wakra Hospital, Hamad Medical Corporation, Doha, Qatar; 3 College of Pharmacy, QU Health, Qatar University, Doha, Qatar; 4 Psychiatry Department, Mental Health Services, Hamad Medical Corporation and College of Medicine Qatar University, Doha, Qatar

**Keywords:** adverse drug reaction, extrapyramidal symptoms, hyperprolactinemia, patient safety, antipsychotic agents, antidepressant agents

## Abstract

**Purpose:**

Adverse Drug Reaction (ADR) reports using spontaneous reporting systems are known to assist in the identification of new, rare, and serious ADRs in clinical practice. Research characterizing ADRs in patients taking psychotropics in Qatar is scarce. This study aims to describe psychotropic-related ADRs’ characteristics, risk factors, severity, causality, and preventability.

**Patients and Methods:**

This is a retrospective chart review of patients within Hamad Medical Corporation’s (HMC) Mental Health Services (MHS) between 2018–2022. ADR reports submitted to the Spontaneous Reporting System database were electronically extracted and analyzed. Data on the type, frequency, onset, severity, and outcomes of adverse reactions, along with patient demographics and comorbidities, were extracted and summarized using quantitative methods.

**Results:**

A total of 908 reports were analyzed. Most ADRs were reported in males (543, 59.83%), in the year 2020 (256, 28.2%). Most reactions were categorized as “moderate” (67.7%), followed by “mild” reactions (30.9%). Extrapyramidal symptoms (EPS) were the highest reported ADRs (46.5%) followed by gastrointestinal side effects (13.3%) and hyperprolactinemia (13.2%) with Haloperidol as the most reported offending agent. Around half of the ADRs were classified as probable and 65.0% were deemed not preventable Psychotropic polypharmacy remained a significant independent risk factor for EPS, increasing the odds of by 45% (OR = 1.45, 95% CI [1.08–1.95], p = 0.015).

**Conclusion:**

This is the first study to describe the characteristics of psychotropic-related ADRs reported within HMC mental health services in Qatar. Most ADRs were moderate in severity with EPS being the most common ADR and haloperidol as the most reported suspected offending agent. Patterns discovered in this study can aid in enhancing patient safety through educating health care providers on predicting and minimizing preventable ADRs.

## Introduction

Psychotropic medications are the cornerstone pharmacological treatment for psychiatric disorders such as schizophrenia, bipolar disorder, and major depressive disorder. They play a vital role in stabilizing mood, reducing psychotic symptoms, and enhancing overall functioning in individuals with severe mental health conditions. However, despite their therapeutic benefits, these agents are associated with a wide range of adverse drug reactions (ADRs), which can compromise patient safety, reduce adherence and negatively impact long-term clinical outcomes.^1^

Among psychotropic medications, antipsychotics are particularly important to consider, as each class and generation carries a distinct ADR profile that can be influenced by patient factors such as age, gender, and genetic background.^2^ First-generation (typical) antipsychotics are closely associated with extrapyramidal symptoms (EPS) such as akathisia, dystonia, and tardive dyskinesia, primarily due to their potent dopamine D2 receptor blockade. In contrast, second-generation (atypical) antipsychotics, while less likely to cause EPS, are frequently associated with metabolic disturbances such as weight gain, dyslipidemia, and diabetes, as well as cardiovascular effects such as QT interval prolongation.^3–5^ The risk and severity of these ADRs may often increase with prolonged use, higher dosages, and the presence of additional risk factors such as comorbidities or polypharmacy.^2^

Similarly, antidepressants are associated with class-specific ADRs that affect tolerability. Antidepressants, including selective serotonin reuptake inhibitors (SSRIs), serotonin-norepinephrine reuptake inhibitors (SNRIs), tricyclic antidepressants (TCAs), and others, are commonly used for mood and anxiety disorders. Typical ADRs include gastrointestinal disturbances (nausea, diarrhea, constipation), insomnia or somnolence, sexual dysfunction, headache, and weight changes.^6^,^7^ Moreover, psychostimulants, used primarily for attention-deficit/hyperactivity disorder (ADHD) and cognitive enhancement, are frequently associated with insomnia, decreased appetite, gastrointestinal disturbances, anxiety, increased heart rate, and elevated blood pressure.^8^ Other psychotropic medications, including anxiolytics, sedatives, and adjunctive antipsychotics, often produce ADRs such as sedation, cognitive slowing, impaired coordination, orthostatic hypotension, and, occasionally, paradoxical agitation. Long-term use of these agents may be associated with tolerance, dependence, or withdrawal phenomena.^9^

ADRs from psychotropic medications can lead to reduced adherence to treatment and therapy discontinuation, resulting in poor clinical outcomes.^10^ The clinical and economic impact of ADRs associated with antipsychotic use is substantial, as these reactions are associated with increased morbidity, mortality, increased healthcare costs and greater resource utilization.^11–13^ Variability in ADR definitions, severity, causality, and preventability presents challenges in accurately assessing their prevalence and the identification of associated risk factors. Recognizing these variations is essential to identify high-risk patients and to implement targeted preventive strategies.

Despite their widespread use, there remains a critical need for comprehensive evaluation of the frequency, severity, and risk factors of ADRs to guide safer prescribing practices. Understanding the relationship between psychotropic medication use and ADRs is crucial for optimizing treatment strategies, minimizing harm, improving adherence and overall outcomes in psychiatric care. With ongoing pharmacovigilance and research, strategies to mitigate these risks continue to evolve, aiming to maximize the therapeutic benefit while minimizing harm. In support of this, recent studies have highlighted the prevalence and clinical impact of these ADRs. A comprehensive analysis of data from the Korean Adverse Event Reporting System database reported 5067 adverse events associated with antipsychotics between 2010 and 2019.^14^ Another study focusing on antipsychotic drug utilization and ADR profiling found that among 250 patients, risperidone (40.3%) and olanzapine (26.3%) were the most frequently prescribed antipsychotics, with most ADRs classified as mild and non-preventable.^15^

In Qatar, the Mental Health Services (MHS) at Hamad Medical Corporation (HMC) serves as the primary facility for prescribing psychotropic medications. It has established mechanisms for monitoring the safety of these agents, primarily through spontaneous reports, which represent the main source of information about ADRs. However, detailed data describing characteristics of ADRs from psychotropic medicines in this population remain scarce. A study by Al Hail et al explored the safety practices and pharmacovigilance in Qatar.^16^ Their findings revealed that in the years 2016 and 2017, a total of 1599 ADRs were reported across the health facilities within HMC, of which only 42 ADRs originated from the MHS. In addition, the majority of the reported ADRs were found to be probable according to the Naranjo’s Scale (49.9%), not preventable (87.9%), mild in severity (47.7%), and reported by pharmacists (57%).^16^ Despite the necessity of characterizing ADRs in patients using psychotropic drugs, evidence on their post-marketing safety in Qatar remains limited.^17^,^18^

The findings of this research could provide valuable insights for mental health clinicians, aiding them in creating more informed and safer treatment decisions for this vulnerable population. The objectives of this study were to describe the characteristics of ADRs associated with psychotropic medications, including their type, frequency, time of onset, and clinical outcomes; to investigate patient- and drug-related risk factors associated with psychotropic-related ADRs; and to evaluate the severity, causality, and preventability of reported ADRs. We hypothesize that ADRs related to psychotropic medications are underreported and that certain psychotropic drug classes and patient characteristics are associated with a higher risk of ADRs.

## Materials and Methods

### Setting

The State of Qatar is a peninsula located on the Arabian Gulf with a population of approximately 2.7 million. Hamad Medical Corporation is the principal provider of government-funded healthcare services in the country and delivers most mental health care through its Mental Health Services. The hospital provides both inpatient and outpatient services to diverse populations, including children, adolescents, adults, and older adults. HMC also serves as the primary facility for prescribing psychotropic medications and has established pharmacovigilance mechanisms to monitor their safety, primarily relying on spontaneous reports as the main source of information regarding ADRs.^19^

### Study Design

This was a cross–sectional quantitative retrospective chart review of patients receiving care at MHS under HMC in Qatar. The primary objective of this study was to describe the characteristics of the ADRs that are associated with the use of psychotropic medications including evaluating their severity, causality, and preventability. Furthermore, to describe the characteristics of the ADRs that occurred with psychotropic medications and investigate the risk factors that are associated with psychotropic-related ADRs.

### Ethics, Consent and Permissions

Ethics approval for conducting this study was received from HMC Review Board (HMC-IRB) (reference number: MRC-01-22-792). No consent was obtained from patients as the nature of the study was retrospective review. Participants were identified by using codes and the link between code and identifier was deleted at the end of the study and the information collected was reported as group data. The study was conducted in accordance with principles of the “Declaration of Helsinki”, Good Clinical Practice (GCP) and within the laws and regulations of Ministry of public health (MOPH) in Qatar.

### Study Participants

All ADRs reports submitted between 2018–2022 by health care professionals to the electronic health records (EHR) system (Cerner system^®^) for patients who are receiving psychiatric care at the facility in both inpatient and outpatient settings were analyzed. Reports have been included if they fulfilled the following criteria: One or more suspected offending agents were identified as psychotropic medications. The study excluded reports submitted where the suspected offending agents were not psychotropic medications. For this study, psychotropic medications were defined using the World Health Organization WHO Anatomical Therapeutic Chemical ATC classification system.^20^ Drugs that fall under nervous system category including N04BD (Monoamine Oxidase B inhibitors; MAO-B inhibitors), N05 (Psycholeptics including antipsychotics, anxiolytics, and sedatives and hypnotics), N06A (Antidepressants), and N06B (Psychostimulants, Agents used for ADHD and Nootropics) were included.

### Data Collection

All ADR reports in which psychotropic medications were suspected to be the offending agent were extracted. The following were collected for each submitted report: the clinical description of the ADR, the suspected offending agent and its dosing regimen, time at which the ADR occurred, time to onset of the ADR after the exposure to the suspected offending agent, the seriousness of the ADR and its outcome(s). All the elements mentioned previously are mandatory fields in the ADR form on Cerner to ensure the completeness of the collected data. Hartwig’s Severity Assessment Scale, Naranjo Algorithm and Schumock and Thornton Criteria were used for Severity, Causality, and preventability assessment, respectively.

Hartwig’s Severity Assessment Scale, the Naranjo Algorithm, and the Schumock and Thornton Criteria are standardized tools used to assess different dimensions of ADRs. Hartwig’s Scale classifies the severity of an ADR based on clinical outcomes and required interventions, ranging from mild (no change in therapy) to severe (life-threatening, requiring intensive care, or resulting in permanent harm or death). The Naranjo Algorithm assesses causality by using a structured questionnaire that assigns a probability score to determine whether the reaction is definite, probable, possible, or doubtful in relation to the suspected drug. The Schumock and Thornton Criteria evaluate preventability by determining whether the ADR could have been avoided through appropriate prescribing, monitoring, dose adjustment, or consideration of patient-specific risk factors. Together, these tools provide a systematic framework to assess the clinical impact, likelihood, and preventability of ADRs in clinical practice and research. The assessment was independently performed by the medication safety officer of the hospital as part of the HMC pharmacovigilance workstreams. ADR classification was based on physician judgment, clinical evaluation, temporal association with psychotropic medication use, and documentation in the medical record. Symptoms that were deemed attributable to underlying medical illness rather than medication effects were not counted as ADRs due to psychotropics by the medication safety officer.

Additionally, the electronic charts of the patients who experienced the reported ADR were reviewed to extract the following information: age, gender, psychiatric diagnosis, duration of illness, number of hospitalizations, concomitant psychotropic medications, other medications, comorbidities, body mass index (BMI), and social history including smoking, alcohol, and illicit drug use.

### Sampling Method and Sample Size Determination

All patients’ records that satisfy the predetermined inclusion and exclusion criteria were included to the study. The study employed a consecutive (census) sampling method; thus, no randomization or sampling was performed. Every eligible patient within the specified cohort and time frame was included, representing the fixed available population.

### Data Analysis

Data collected in the data collection form were entered into Stata v.18.0 SE (StataCorp, College Station, TX, USA) to perform descriptive and inferential statistical analysis. Descriptive statistics, such as the mean ± standard deviation (SD), median and interquartile ranges (IQR), and frequencies expressed in number (n) and percentage (%), were used. Normality testing was performed using the Shapiro–Wilk test p-value as a numerical output. Depending on the distribution and nature of the variables, categorical variables were compared using the Chi-square test (or Fisher’s exact test when applicable), with effect sizes reported using Cramér’s V, and the Mann–Whitney *U*-test for continuous variables. To identify independent predictors of ADRs while adjusting for potential confounders, multivariable logistic regression analyses were performed, and results were expressed as odds ratios (ORs) with corresponding 95% confidence intervals (CIs). Statistical significance was predetermined at a p≤0.05.

## Results

### General Characteristics of Spontaneous Reports and Patient Population

**Figure 1:**
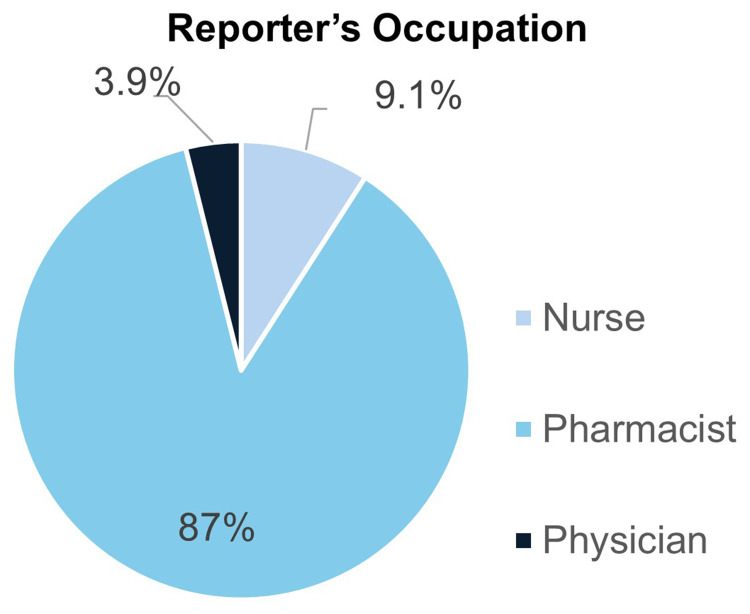
ADR Reports by Reporter’s Profession.

**Figure 2:**
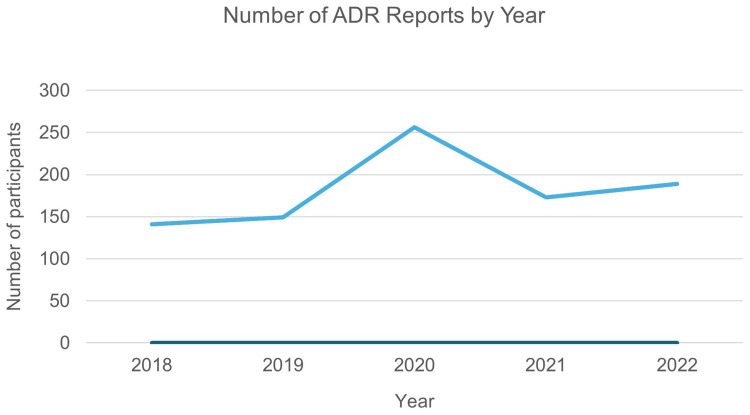
Annual Trend of ADR Reports, 2018–2022.

This retrospective analysis was based on a total of 908 spontaneous Adverse Drug Reaction (ADR) reports associated with at least one psychotropic medication, collected by the mental health services in Qatar’s HMC between January 1, 2018, and November 29, 2022. An evaluation of the reporters’ professions revealed that pharmacists submitted the majority of reports (n=790, 87.0%), followed by nurses (n=83, 9.1%) (Figure 1). The year 2020 had the most ADRs, with 28.2% (n=256) of all reports for the study period (Figure 2). Moreover, Table 1 shows a full demographic and clinical profile of the 908 patients who had these ADRs. The patient group was mostly male, with 59.8% (n=543) of the reports being from males and 40.2% (n=365) from females. The age distribution indicated that the reactions were predominantly reported among young to middle-aged adults. The largest group was patients between the ages of 26 and 35, who made up 36.9% (n=335) of the population. The second largest group was patients between the ages of 18 and 25, who made up 24.2% (n=219). Patients in the 36–45-year range made up 21.1% (n=192) of the cohort. Reports for pediatric (<18 years) and geriatric (>65 years) patients were less common, at 2.6% and 1.8%, respectively. The majority of the patients were non-Qatari (61.6%, n=559). A large proportion of patients (56.9%, n=516) were from the Middle East and North Africa (MENA) region, which was followed by South Asia (21.7%, n=197) and Sub-Saharan Africa (15.1%, n=137) according to the World Bank’s definition of geographical regions. Additionally, the majority of patients (93.2%, n=846) had no known history of allergies. Clinical history revealed that most patients were not new to psychotropic treatment, with 88.9% (n=807) having had prior exposure. Additionally, more than a third of the cases (37.6%, n=341) showed psychotropic polypharmacy. The patient cohort was predominantly defined by a history of psychiatric hospitalization, with 93.0% (n=844) having experienced prior admission.

**Table 1:** Patient Demographic and Clinical Characteristics (N=908)

Parameter		n (%)
Gender	Males	543 (59.8%)
	Females	365 (40.2%)
Age	<18	24 (2.6%)
	18-25	219 (24.2%)
	26-35	335 (36.9%)
	36-45	192 (21.1%)
	46-55	87 (10.1%)
	56-65	35 (3.3%)
	>65	16 (1.8%)
Nationality	Qatari	349 (38.4%)
	Non-Qatari	559 (61.6%)
Nationality (Grouped by geographical region)	Middle East and North Africa (MENA)	516 (56.9%)
	South Asia	197 (21.7%)
	Sub-Saharan Africa	137 (15.1%)
	East Asia and the Pacific	32 (3.5%)
	Europe and Central Asia	16 (1.8%)
	North America	8 (0.9%)
	Latin America and the Caribbean	1 (0.1%)
	Unspecified	1 (0.1%)
Allergy	Know allergy	62 (6.8%)
Naïve to psychotropics	Naïve to psychotropic	101 (11.1%)
Psychotropics polypharmacy	Polypharmacy	341 (37.6%)
Number of Hospitalizations	0	64 (7.0%)
	1	358 (39.4%)
	2	207 (22.8%)
	3-4	184 (20.3%)
	5+	95 (10.5%)
Admission	First-time admission	64 (7.0%)
	Previous admission	844 (93.0%)
Medical comorbidities	Known comorbidities	215 (23.7%)
	Metabolic and endocrine disorders	141 (15.5%)
	Neurological disorders/stroke	12 (1.3%)
	Respiratory disorders	26 (2.9%)
	Cardiovascular disorders/blood pressure	69 (7.6%)
	Gastrointestinal disorders	18 (2%)
	Renal disorders	11 (1.2%)
History of smoking	Current	230 (25.3%)
	Previous	29 (3.2%)
	Never smoked	440 (48.5%)
	Unable to identify	209 (23.0%)
History of illicit drug use	Yes	124 (13.7%)
	No	555 (61.1%)
	Unable to know	229 (25.2%)
History of alcohol	Yes	113 (12.4%)
	No	592 (65.2%)
	Unable to know	203 (22.4%)
Body Mass Index (BMI)	Healthy weight	324 (35.7%)
	Underweight	28 (3.1%)
	Overweight	251 (27.6%)
	Obesity	305 (33.6%)

As for physical health, many patients did not have documented medical comorbidities (76.3%, n=693). However, among the 23.7% (n=215) who did, metabolic and endocrine disorders were the most common type of comorbidity, which presented in 15.5% (n=141) of the total patient population. The next most frequent comorbidity was cardiovascular disorders, which were present in 7.6% (n=69) of the patients. There were fewer reports of other comorbidities, such as neurological, gastrointestinal, respiratory, and renal conditions. BMI data show that weight problems are prevalent in this community, with a third of patients (33.6%, n=305) meeting the criteria for obesity and another 27.6% (n=251) being overweight. Of the total population, only 35.7% met the criteria for healthy weight range. Moreover, social history data showed that 25.3% (n=230) were current smokers, in addition to 13.7% (n=124) had a history of illicit drug use, and 12.4% (n=113) reported alcohol consumption.

**Figure 3:**
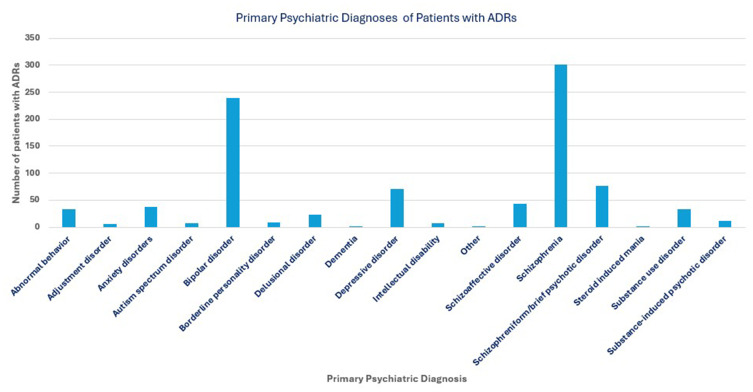
Primary Psychiatric Diagnoses of Patients with ADRs.

**Figure 4:**
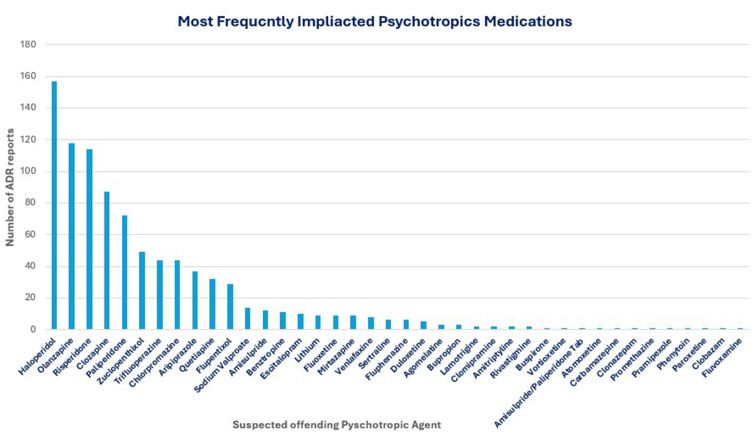
Most Frequently Implicated Psychotropic Medications.

The primary psychiatric diagnoses associated with the ADR reports are presented in Figure 3. The spectrum was dominated by psychotic and mood disorders with schizophrenia being the most common diagnosis, accounting for 33.1% (n=301) of diagnoses. Bipolar disorder was the second most reported diagnosis at 26.4% (n=240). Other psychotic disorders, such as schizophreniform or brief psychotic disorder, were noted in 8.5% (n=77) of cases, while depressive disorders were present in 7.8% (n=71). The medication most frequently suspected as the offending agent was haloperidol at 17.3%. Followed by olanzapine (13.0%), risperidone (12.6%), and paliperidone (7.9%) as in Figure 4.

**Figure 5:**
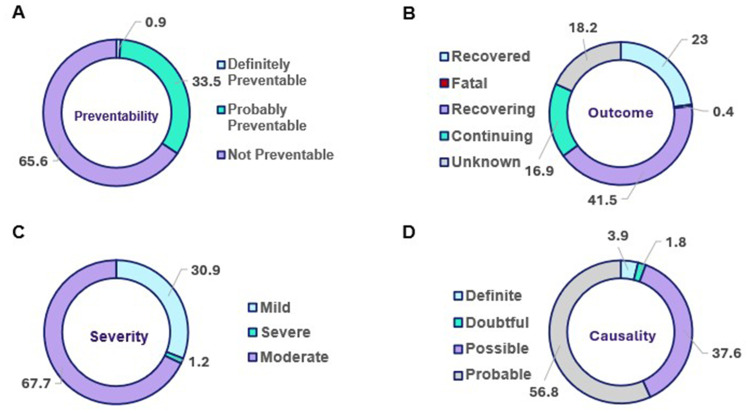
Clinical Assessment of Reported ADRs. (**A**) The Schumock and Thornton Preventability Criteria assesses preventability by identifying whether the ADR could have been avoided through appropriate drug selection, dosing, monitoring, or consideration of patient-specific risk factors, (**B**) Clinical Outcomes documented, (**C**) The Hartwig Severity Assessment Scale categorizes ADR severity based on clinical outcomes and the level of intervention required, ranging from mild reactions that do not necessitate a change in therapy to severe events that are life-threatening, require intensive care, or result in permanent disability or death and (**D**) The Naranjo Adverse Drug Reaction Probability Scale determines causality through a structured questionnaire that generates a probability score, classifying the relationship between the suspected drug and the reaction as definite, probable, possible, or doubtful.

The clinical nature of the ADRs was revealed by an evaluation of the ADRs themselves as in Figure 5. Most reactions were classified as “not preventable” (65.0%) based on the modified Schumock and Thornton criteria; however, a sizable percentage were classified as “probably preventable” (33.5%). Almost two thirds of reactions were categorized as “moderate” (67.7%), followed by “mild” reactions (30.9%) and “severe” reactions (1.2%). According to the Naranjo causality evaluation, the most frequent association between the drug and the reaction was “probable” (56.8%), which was followed by “possible” (37.6%). Many reactions remained unresolved in terms of clinical outcomes at the time of reporting; 41.5% of patients were still experiencing the ADR (“ongoing”), and 23.0% were “recovering”. While only 0.4% of reports showed fatal results, compared to 18.2% of cases with full resolution.

### Analysis of Specific Adverse Drug Reactions

**Figure 6:**
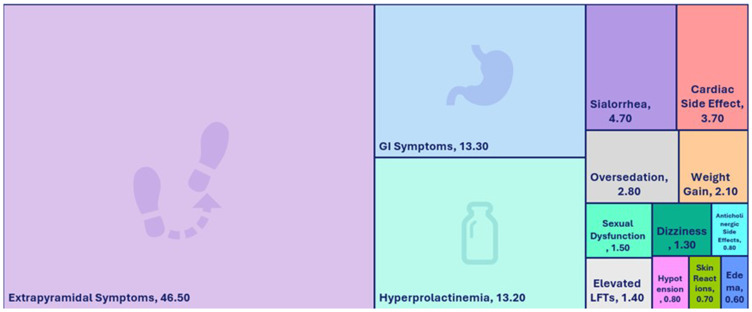
Distribution of Adverse Drug Reaction Types Associated with The Use of Psychotropic Medications (%).

To determine the clinical and patient characteristics linked to the three most reported ADRs (EPS, hyperprolactinemia and GI symptoms) in Figure 6, a detailed statistical analysis was carried out.

#### Extrapyramidal Symptoms (EPS)

With 46.5% of all complaints, EPS was the most common ADR. To find correlations between different characteristics and the emergence of EPS, a bivariate analysis was conducted (Table 2). Gender was shown to be statistically significant, with a minor to moderate effect size (Cramér’s V = 0.169) and a larger percentage of males developing EPS (53.4%) compared to females (36.2%) (χ^2^(1) = 26.09, p < 0.001). Another important predictor was the use of numerous psychotropic drugs, or polypharmacy, with 52.2% of patients experiencing EPS (χ^2^(1) = 7.19, p = 0.007, Cramér’s V = 0.089). Lifestyle factors also had a role; current smokers had the highest frequency of EPS (55.7%), and smoking status was significantly correlated with EPS (χ^2^(3) = 10.72, p = 0.013). A history of illicit drug use (χ^2^(2) = 25.06, p < 0.001) and alcohol usage (χ^2^(2) = 15.02, p < 0.001) were also significantly linked to an increased incidence of EPS. Additionally, a history of illicit drug use (χ^2^(2) = 25.06, p < 0.001) was also strongly associated with a higher incidence of EPS. In contrast, being naïve to psychotropic medication was not significantly associated with EPS development (p = 0.436). Furthermore, none of the assessed medical comorbidity categories showed a statistically significant relationship with EPS. The distribution of continuous variables, age and BMI, did not differ significantly between patients who developed EPS and those who did not, as determined by the Mann–Whitney *U*-test (Table 3).

**Table 2:** Bivariate Analysis of Factors Associated with Common ADRs

Variables	Developing Hyperprolactinemia					Developing EPS					Developing GI Symptoms				
	Hyper-PRL n (%)	No Hyper-PRL n (%)	χ^2^ (df)	p-value	Cramér’s V	EPS n (%)	No EPS n (%)	χ^2^ (df)	p-value	Cramér’s V	GI n (%)	No GI n (%)	χ^2^ (df)	p-value	Cramér’s V
**Gender**			53.98 (1)	**<0.001**	0.244			26.09 (1)	**<0.001**	0.169			5.12 (1)	**0.024**	0.075
Male	35 (6.4)	508 (93.6)				290 (53.4)	253 (46.6)				61 (11.2)	482 (88.8)			
Female	85 (23.3)	280 (76.7)				132 (36.2)	233 (63.8)				60 (16.4)	305 (83.6)			
**Psychotropic Polypharmacy**			3.37 (1)	0.067	0.061			7.19 (1)	**0.007**	0.089			0.84 (1)	0.358	0.031
Yes	36 (10.6)	305 (89.4)				178 (52.2)	163 (47.8)				50 (14.7)	291 (85.3)			
**Naïve to Psychotropics**			2.17 (2)	0.338	0.049			1.66 (2)	0.436	0.043			11.27 (2)	**0.004**	0.111
Yes	9 (8.9)	92 (91.1)				53 (52.5)	48 (47.5)				23 (22.8)	78 (77.2)			
**Medical Comorbidities (Any)**			1.56 (1)	0.212	0.041			0.23 (1)	0.630	0.016			0.14 (1)	0.705	0.013
Yes	23 (10.7)	192 (89.3)				103 (47.9)	112 (52.1)				27 (12.6)	188 (87.4)			
**Metabolic/Endocrine Disorder**			4.27 (1)	**0.039**	0.069			0.41 (1)	0.524	0.021			0.11 (1)	0.744	0.011
Yes	11 (7.8)	130 (92.2)				69 (48.9)	72 (51.1)				20 (14.2)	121 (85.8)			
**Neurological Disorder/Stroke**			0.13 (1)	0.722	0.012			0.11 (1)	0.737	0.011			1.43 (1)	0.231	0.040
Yes	2 (16.7)	10 (83.3)				5 (41.7)	7 (58.3)				3 (25.0)	9 (75.0)			
**Cardiovascular Disorder**			0.61 (1)	0.433	0.026			0.05 (1)	0.815	0.008			0.09 (1)	0.767	0.010
Yes	7 (10.1)	62 (89.9)				33 (47.8)	36 (52.2)				10 (14.5)	59 (85.5)			
**Gastrointestinal Disorder**			0.94 (1)	0.332	0.032			1.58 (1)	0.209	0.042			2.82 (1)	0.093	0.056
Yes	1 (5.6)	17 (94.4)				11 (61.1)	7 (38.9)				0 (0.0)	18 (100.0)			
**Renal Disorder**			1.70 (1)	0.193	0.043			0.005 (1)	0.946	0.002			0.23 (1)	0.634	0.016
Yes	0 (0.0)	11 (100.0)				5 (45.5)	6 (54.5)				2 (18.2)	9 (81.8)			
**Smoking Status**			14.46 (3)	**0.002**	0.126			10.72 (3)	**0.013**	0.109			22.30 (3)	**<0.001**	0.157
Never Smoked	68 (15.5)	372 (84.5)				190 (43.2)	250 (56.8)				80 (18.2)	360 (81.8)			
Ex-smoker	1 (3.4)	28 (96.6)				14 (48.3)	15 (51.7)				6 (20.7)	23 (79.3)			
Current Smoker	16 (7.0)	214 (93.0)				128 (55.7)	102 (44.3)				22 (9.6)	208 (90.4)			
Unable to identify	35 (16.7)	174 (83.3)				90 (43.1)	119 (56.9)				13 (6.2)	196 (93.8)			
**Alcohol Use**			7.02 (2)	**0.030**	0.088			15.02 (2)	**<0.001**	0.129			17.54 (2)	**<0.001**	0.139
Yes	12 (10.6)	101 (89.4)				70 (61.9)	43 (38.1)				10 (8.8)	103 (91.2)			
**Illicit Drug Use**			19.91 (2)	**<0.001**	0.148			25.06 (2)	**<0.001**	0.166			14.56 (2)	**<0.001**	0.127
Yes	2 (1.6)	122 (98.4)				83 (66.9)	41 (33.1)				10 (8.1)	114 (91.9)			

**Notes**: Data are presented as count (n) and column percentage (%). The association between categorical variables was assessed using the Pearson’s Chi-square (χ^2^) test. Cramér’s V is reported as a measure of effect size. A p-value < 0.05 was considered statistically significant and are in bold.

**Abbreviations**: df, degrees of freedom; EPS, extrapyramidal symptoms; GI, gastrointestinal; Hyper-PRL, hyperprolactinemia.

**Table 3:** Non-Parametric Comparison of Age and BMI Across Key ADRs

Variable	Group	N (Obs)	Mean Rank	z-statistic	p-value
**Age**				1.441	0.150
	No HyperPRL	788	459.4		
	HyperPRL	120	422.4		
				0.592	0.554
	No EPS	486	459.3		
	EPS	422	449.0		
				−0.944	0.345
	No GI Symptom	787	451.3		
	GI Symptom	121	475.4		
**BMI**				−0.095	0.924
	No HyperPRL	708	405.7		
	HyperPRL	103	408.0		
				1.131	0.258
	No EPS	422	415.0		
	EPS	389	396.3		
				−0.440	0.660
	No GI Symptom	701	404.6		
	GI Symptom	110	415.1		

**Notes**: The Mann–Whitney *U*-test was used to compare the distribution of age and BMI between groups. Data presented are the number of observations (N), mean rank, and z-statistic. A p-value < 0.05 was considered statistically significant (none was significant).

**Abbreviations**: BMI, body mass index; EPS, extrapyramidal symptoms; GI, gastrointestinal; HyperPRL, hyperprolactinemia; N (Obs), number of observations.

To identify independent predictors while controlling other variables, a multivariable logistic regression was performed (Table 4). The model revealed that being female was independently associated with 48% lower odds of developing EPS compared to being male (OR = 0.52, 95% CI [0.39–0.71], p < 0.001). Psychotropic polypharmacy remained a significant independent risk factor, increasing the odds of developing EPS by 45% (OR = 1.45, 95% CI [1.08–1.95], p = 0.015). Age was also a significant predictor, with patients under 18 years of age having 67% lower odds of EPS compared to the 18–25 reference group (OR = 0.33, 95% CI [0.12–0.94], p = 0.038). Finally, certain psychiatric diagnoses were associated with significantly lower odds of EPS compared to the reference diagnosis of substance-induced psychotic disorder. Specifically, patients with obsessive-compulsive and related disorders had 92% lower odds (OR = 0.08, 95% CI [0.01–0.88], p = 0.039), and those with other anxiety disorders had 90% lower odds (OR = 0.10, 95% CI [0.02–0.58], p = 0.010) of experiencing EPS.

**Table 4:** Multivariable Logistic Regression of Predictors for EPS

Variable	Odds Ratio (OR)	Std. Err.	z	p-value	[95% Conf. Interval]
**Age Group (Ref: 18–25)**					
26-35	0.87	0.17	−0.73	0.467	0.60–1.26
36–45	1.01	0.22	0.06	0.950	0.66–1.56
46–55	1.08	0.30	0.26	0.796	0.62–1.86
56-65	1.14	0.44	0.35	0.726	0.54–2.42
< 18	0.33	0.18	−2.08	**0.038**	0.12–0.94
> 65	0.24	0.19	−1.83	0.068	0.05–1.11
**Gender (Ref: Male)**					
Female	0.52	0.08	−4.25	**<0.001**	0.39–0.71
**Psychotropic Polypharmacy (Ref: No)**					
Yes	1.45	0.22	2.44	**0.015**	1.08–1.95
**Naïve to Psychotropics (Ref: No)**					
Yes	1.55	0.38	1.77	0.076	0.96–2.51
Unable to know	1.17	1.67	0.11	0.910	0.07–19.18
**Psychiatric Diagnosis (Ref: Substance‐induced psychotic disorder)**					
Adjustment disorder	0.60	0.63	−0.49	0.625	0.08–4.74
Autism spectrum disorder	0.59	0.61	−0.51	0.611	0.08–4.43
Bipolar disorder	0.63	0.42	−0.69	0.488	0.18–2.29
Delusional disorder	0.28	0.22	−1.61	0.107	0.06–1.32
Dementia	2.77	4.82	0.59	0.558	0.09–83.91
Depressive disorder	0.37	0.26	−1.44	0.150	0.10–1.43
Intellectual disability	4.91	6.27	1.25	0.212	0.40–59.90
OCD and related disorders	0.08	0.10	−2.06	**0.039**	0.01–0.88
Other	0.49	0.35	−1.00	0.319	0.12–2.01
Other anxiety disorders	0.10	0.09	−2.58	**0.010**	0.02–0.58
Personality disorder	3.76	4.75	1.05	0.295	0.32–44.73
Schizoaffective disorder	0.99	0.71	−0.02	0.986	0.24–4.03
Schizophreniform/brief psychotic	0.45	0.31	−1.16	0.245	0.12–1.72
Substance use disorder	2.53	1.98	1.19	0.236	0.55–11.71

**Notes**: The model predicts the odds of developing extrapyramidal symptoms. Reference categories for variables are indicated in parenthesis (e.g., “Ref: Male”). A p-value < 0.05 was considered statistically significant and are in bold.

**Abbreviations**: Conf. Interval, Confidence Interval; OR, Odds Ratio; Ref, Reference Category; Std. Err., Standard Error.

#### Hyperprolactinemia

Hyperprolactinemia was reported in 13.2% of cases as in Figure 4. The bivariate analysis (Table 2) revealed a strong and statistically significant association with gender (χ^2^(1) = 53.98, p < 0.001), with a much higher proportion of females (23.3%) experiencing this ADR compared to males (6.4%). The effect size was moderate (Cramér’s V = 0.244). The presence of a pre-existing metabolic or endocrine disorder was also a significant, though weak, factor (χ^2^(1) = 4.27, p = 0.039). Lifestyle factors were also significant; smoking status showed a complex association (χ^2^(3) = 14.46, p = 0.002), with the highest incidence among those who had never smoked (15.5%) and the lowest among ex-smokers (3.4%). Alcohol use (χ^2^(2) = 7.02, p = 0.030) and a history of illicit drug use (χ^2^(2) = 19.91, p < 0.001) were also significantly associated, with illicit drug use being linked to a lower incidence of hyperprolactinemia. Factors such as psychotropic polypharmacy (p=0.067) and being naïve to psychotropics (p=0.338) were not significantly associated. The Mann–Whitney *U*-test showed no significant difference in the distribution of age or BMI for patients who developed hyperprolactinemia compared to those who did not (Table 3).

#### Gastrointestinal (GI) Symptoms

GI symptoms were reported in 13.3% of cases (Figure 4). The bivariate analysis (Table 2) found a significant association with gender, with females experiencing a higher incidence (16.4%) compared to males (11.2%) (χ^2^(1) = 5.12, p = 0.024). Being naïve to psychotropic medications was also a significant factor, linked to a notably higher incidence of GI symptoms (22.8%) compared to those with prior exposure (χ^2^(2) = 11.27, p = 0.004). Lifestyle factors were all strongly associated with the development of GI symptoms, including smoking status (χ^2^(3) = 22.30, p < 0.001), alcohol use (χ^2^(2) = 17.54, p < 0.001), and illicit drug use (χ^2^(2) = 14.56, p < 0.001). Psychotropic polypharmacy and the presence of any medical comorbidities were not significantly associated with GI symptoms. Again, age and BMI were not found to be significant distinguishing factors based on the Mann–Whitney *U*-test (Table 3).

## Discussion

In this study, we described the characteristics of ADRs associated with the use of psychotropic medications in patients receiving care at HMC’s mental health services in Qatar over a five-year period. Adverse drug reactions (ADRs) are varied and commonly encountered, yet they are not often evaluated in a systematic manner.^21^ The study population represented diverse cultural backgrounds, with no single predominant ethnicity. Approximately 89% of the patients had prior exposure to antipsychotic medications, indicating that the majority were not antipsychotic-naïve. More than one-third of the sample was receiving antipsychotic polypharmacy. Similarly, results of 53 studies showed that antipsychotic polypharmacy was associated with increased frequency of adverse effects consistent with our findings.^21^

Our data showed that over half of the reported ADRs occurred in young adults. The German drug surveillance program reported that the likelihood of experiencing ADRs was similar for both older and younger patients. However, older patients in their studies had a notably lower risk of acute dystonia, akathisia, liver dysfunction, weight gain, sexual dysfunction, and hyperprolactinemia. Additionally, they had a higher risk for delirium, orthostatic syncope, and hyponatremia compared to young adult patients. They were also more likely to experience a greater risk of ADRs when multiple drugs were involved.^22^

Approximately 75% of the patients did not have any recorded medical comorbidities, suggesting that the majority were free from medical diseases. This is somewhat unexpected, as chronic medical conditions like obesity, diabetes, and hypertension are commonly found in psychiatric patients, increasing the risk of significant drug interactions and other complications from psychotropic medications.^23^ For instance, in a cross-sectional analysis of approximately 1.7 million patients with bipolar disorders in Scotland, 63.9% had at least one comorbidity.^24^ However, compared to our cohort, the study population was relatively older, with only 27.3% being 44 years old or younger. In contrast, 82.2% of our cohort was 45 years old or younger. Other possible explanations include the incomplete capture or reporting of physical comorbidities in patients’ electronic health records (eg., incomplete problem lists) due to the fact that this could be the first presentation of the patients to medical services, which may lead to an underestimation of comorbidities in retrospective analyses. Of note, our findings are in line with a previous retrospective cohort study conducted in 2017 in our setting.^25^ In this study, investigating medical comorbidities in patients attending outpatient services in the mental health hospital, one-third of patients with serious mental illness had at least one comorbidity.

Haloperidol and Olanzapine, followed by risperidone/Paliperidone were the most frequently reported suspected offending agents to cause ADRs. This is consistent with medications reported in the existing literature. Ayani et al have identified that antipsychotics were associated with half of all reported adverse drug events^26^ Guo et al have reported Haloperidol as the most suspected offending agent from first generation antipsychotics and Risperidone followed by Clozapine and Olanzapine as the most offending agents from the second generations antipsychotics.^27^

The frequency of reporting adverse drug reactions (ADRs) from antidepressants was low. For instance, in an exploration of psychotropic medications ADRs using Portuguese pharmacovigilance data, antidepressants were associated with the highest safety reports (62%).^28^ This difference could possibly be attributed to the relatively low prevalence of depression in our cohort (7.6%) compared to the prevalence in Portugal (12.2%), which is considered the highest in Europe.^29^

Moreover, attitudes towards reporting these adverse reactions remain the primary reasons for underreporting. Although these factors can potentially be altered through educational efforts, it is essential to see changes take place.^30^ Many healthcare professionals may experience uncertainty regarding whether medication is responsible for a particular incident, which may lead them to refrain from reporting it. A deficiency in knowledge or training concerning the ADR-reporting system, such as the misconception that only severe or unknown ADRs require reporting, or the presumption that well-tolerated marketed drugs are inherently safe, exacerbates this issue. Moreover, as ADR reporting was predominantly pharmacist-led, targeted efforts to improve reporting engagement across disciplines may allow for capturing more ADRs. Nonetheless, mental health care is often delivered by multidisciplinary teams. Thus, these findings do not necessarily imply limited involvement of other disciplines in ADR recognition or management but could simply reflect a workflow where reporting responsibilities are delegated to pharmacy services.

When considering the preventability and severity of the reported ADRs, our findings are not significantly different from those in other countries. According to Alshehri et al, in a similar setting in England, one-fifth of the reported ADRs were deemed preventable, at 191%.^31^ In our case, 33% were probably preventable, and less than 1% were definitely preventable Furthermore, approximately 61.7% of the reported ADRs in England were of at least moderate clinical severity, whereas in our data, 67.7% were classified as moderate in severity.^31^

When it comes to the frequency of reported ADRs, EPS and GI symptoms were the most common. The results are consistent with a study in China, which showed that nervous system disorders were almost 47% and GI, which is the second, was about 12% of the reported ADRs. Cardiac disorders came as the fourth ADRs.^27^ It is also consistent with the most commonly suspected medication in our cohort: haloperidol, olanzapine, followed by risperidone and paliperidone. Haloperidol is a high-potency first-generation antipsychotic with high D2 receptor occupancy. It is commonly associated with high rates of acute dystonia, Parkinsonism, and akathisia in comparative studies.^32–35^ Among second-generation agents, Risperidone and its metabolite Paliperidone, as well as Olanzapine, although to a lesser extent, also exhibit dose-dependent EPS.^33^ Moreover, Olanzapine and Risperidone are also associated with GI side effects such as constipation due to their dopaminergic and anticholinergic effects.^35^ This highlights the need for careful agent selection, dose optimization, and practice monitoring of both motor and bowel functions in patients using these high-risk antipsychotics.

In-depth analysis of the factors associated with the most frequently reported ADRs, specifically EPS, reveals that male patients, those with psychotropic polypharmacy, and individuals aged 18–25 years exhibit a higher likelihood of experiencing EPS ADRs. Notably, current smokers demonstrate the highest frequency of EPS. All categories of psychotropic drugs, including anxiolytics, hypnotics, antidepressants, and antipsychotics, show a significant association with EPS, with a stronger tendency observed in cases of polypharmacy involving these medications.^36^ EPS, though associated with antipsychotics, are infrequent yet significant adverse effects linked to the use of antidepressants.^37^ These findings should be interpreted cautiously, given the retrospective design and the reliance on spontaneous reporting.

The connections we observed for gastrointestinal symptoms occurrence and other mentioned factors in our study emphasize the necessity for additional research to elucidate the underlying mechanisms and contributing factors. Frequently prescribed antidepressants exhibit varying gastrointestinal side effect profiles, which may be linked to their mechanisms of action. Clinicians should consider the unique tolerability profile of each medication when prescribing antidepressants to enhance treatment adherence and improve outcomes for patients with mental illness.^38^ Several practical measures can be taken, with early detection, careful drug selection, and supportive care being the primary strategies for prevention.

In this study, although it was a weak risk factor, pre-existing metabolic and endocrine disorders have a significant association. Alosaimi et al noted that in a multivariate analysis, hyperprolactinemia showed an independent and positive correlation with the use of antipsychotic medications and that diabetes, and hypothyroidism were positively linked.^39^

The results of this project highlight the importance of continuous monitoring of ADRs to improve patient safety and optimize treatment outcomes. By identifying the risk factors linked to ADRs over several years, our study lays the groundwork for targeted interventions focused on prevention and early detection. As a subsequent step, it is advised to implement structured trigger tools, especially in inpatient settings, to systematically identify ADRs that might otherwise remain unreported. Trigger tools, such as laboratory abnormalities (eg., elevated liver enzymes, abnormal prolactin levels) or the use of antidotes, act as objective indicators that can prompt clinicians to investigate potential medication-related harm. Evidence indicates that these tools significantly enhance ADR detection rates compared to spontaneous reporting systems alone.^40^,^41^ The continuation of the regular medication safety reviews by medication safety officers can further improve the sensitivity of ADR detection and foster learning across the clinical team.^42^ Educational initiatives should also be undertaken to raise awareness and ensure the consistent use of ADR forms in daily practice. For future research, larger multicenter studies are needed to validate and adapt trigger tools to psychiatric and other specialized settings, where symptom overlaps and polypharmacy often complicate ADR identification. Ultimately, combining traditional pharmacovigilance with proactive detection systems like trigger tools can establish a more comprehensive framework for preventing medication-related harm and enhancing patient safety outcomes.

Our findings support more practical and structured monitoring that is integrated within routine psychiatric care. The high proportion of EPS, hyperprolactinemia, and GI side effects among the reported ADRs in our study, and their association with certain patient characteristics such as those on polypharmacy, suggest that some patient groups might benefit from targeted side-effect surveillance. Clinicians may consider actively assessing these ADRs using brief checklists, especially after initiation and major dose changes.

The study faced limitations due to its retrospective design and dependence on reported ADR data, which might have resulted in underreporting or incomplete documentation. Given the broad service structure and the inclusion of both inpatient and outpatient settings (including community services), it was not feasible to accurately estimate the total number of patient charts or encounters within the study timeframe. Furthermore, the study focused specifically on reported ADRs rather than all patient records. ADR cases were therefore identified through available reporting systems and clinical documentation, and all eligible ADR reports within the study period were included. Consequently, a comprehensive census of ADR cases was performed rather than sampling, and no predefined sample size estimation was applied. This study did not examine the relationship between medication dosing and the occurrence of ADRs. Regional differences in pharmacogenetics may influence drug metabolism and susceptibility to adverse effects, suggesting that dosing strategies used elsewhere might not fully reflect the optimal approach for the local population. Future research exploring the impact of pharmacogenetic variation on ADR risk and dose–response relationships in Qatar could provide valuable guidance for individualized and safer psychotropic prescribing.

## Conclusion

This study provides the first detailed characterization of adverse drug reactions associated with psychotropic medications in Qatar. Antipsychotics were the most frequently implicated, while ADRs for other psychotropic classes may be under-recognized due to limited reporting and the absence of systematic screening. ADR reporting was predominantly pharmacist-led, highlighting possible gaps in physician engagement, particularly for known or expected reactions.

Medications such as clozapine and lithium received greater attention due to the presence of established prescribing and monitoring guidelines. These findings underscore the importance of implementing routine, structured ADR monitoring across all psychotropic classes, enhancing pharmacovigilance, and considering region-specific factors such as genetics and prescribing practices. Future research should explore dose–response relationships, pharmacogenetic influences, and strategies to improve ADR detection and reporting, ultimately supporting safer and more effective psychotropic prescribing in Qatar and the broader Arab region.
